# Peripapillary Vessel Density and Retinal Nerve Fiber Layer Thickness in Patients with Unilateral Primary Angle Closure Glaucoma with Superior Hemifield Defect

**DOI:** 10.5005/jp-journals-10078-1247

**Published:** 2019

**Authors:** Tarannum Mansoori, Nagalla Balakrishna

**Affiliations:** 1 Department of Glaucoma, Sita Lakshmi Glaucoma Center, Anand Eye Institute, Hyderabad, Telangana, India; 2 Department of Statistics, National Institute of Nutrition, Hyderabad, Telangana, India

**Keywords:** OCT angiography, Primary angle-closure, Primary angle-closure glaucoma, Retinal nerve fiber layer thickness, RPC vessel density, Superior hemifield loss

## Abstract

**Purpose:**

To evaluate peripapillary retinal nerve fiber layer (RNFL) thickness and radial peripapillary capillary (RPC) vessel density (VD) in the eyes with unilateral primary angle-closure glaucoma (PACG) with the visual field (VF) defect confined to the superior hemifield and compare these parameters with the corresponding perimetrically intact regions of the fellow eye with primary angle-closure (PAC) and normal control eyes, using optical coherence tomography angiography (OCTA).

**Materials and methods:**

This prospective, cross-sectional study included 28 eyes with unilateral PACG, with VF defects restricted to the superior hemifield, 28 fellow eyes with PAC, and 30 age-matched normal controls. Peripapillary RNFL thickness and RPC VD were measured in the eight peripapillary sectors, using OCTA, and these parameters were compared among the corresponding sectors of PACG, PAC, and healthy eyes using analysis of variance (ANOVA) with the Bonferroni *post hoc* analysis.

**Results:**

In PACG eyes, there was a significant difference in the RNFL thickness (*p* < 0.0001) and RPC VD (*p* = 0.001) between the superior and the inferior hemifield. In PAC and normal eyes, there was no significant difference in the RNFL thickness and RPC VD between the superior and the inferior hemifield. Within the perimetrically intact regions of the PACG eyes, the mean RNFL thickness was significantly reduced in the superonasal (SN) and upper nasal (UN) sectors (*p* = 0.02), but the VD did not show any significant difference, when compared to the fellow PAC eyes. In PACG eyes, the mean RNFL thickness was significantly reduced in the perimetrically normal SN and UN sectors (*p* < 0.0001) and the VD was reduced in the UN sector (*p* = 0.01), when compared to the normal eyes. When comparing the peripapillary sectors of the PAC and healthy eyes, RNFL thickness was reduced in UN (*p* = 0.02), lower nasal (LN) (*p* = 0.01), inferonasal (IN) (*p* = 0.02), and inferotemporal (IT) sectors (*p* = 0.03) and there was no significant difference in the VD in any of the sectors. Inside disc capillaries were preserved in all the three groups.

**Conclusion:**

Sector-wise RNFL thinning seems to precede the vascular changes and functional loss in the PAC and PACG eyes.

**How to cite this article:**

Mansoori T, Balakrishna N. Peripapillary Vessel Density and Retinal Nerve Fiber Layer Thickness in Patients with Unilateral Primary Angle Closure Glaucoma with Superior Hemifield Defect. J Curr Glaucoma Pract 2019;13(1):21–27.

## INTRODUCTION

Glaucoma is a multifactorial optic neuropathy characterized by a progressive retinal ganglion cell loss and glaucomatous visual field (VF) defect. In glaucoma, the structural damage may precede functional deficit, as seen in the white on white standard automated perimetry (SAP).^1^ Decreased optic nerve head (ONH) perfusion and peripapillary vessel density (VD) has been reported in primary open-angle glaucoma (POAG) and primary angle-closure glaucoma (PACG).^2–5^

RPCs are the most superficial of the capillary layers, seen between the internal limiting membrane (ILM) and the RNFL.^6^ Optical coherence tomography angiography (OCTA) imaging with RTVue-XR 100 Avanti OCT (AngioVue, AngioAnalytics, Optovue Inc., Fremont, CA) has a three-dimensional orthogonal registration algorithm called the split spectrum amplitude-decorrelation angiography (SSADA) for imaging and quantification of peripapillary RPC and ONH microcirculation.^5,7^ Studies with OCTA have demonstrated reduced ONH and peripapillary VD in the patients with glaucoma.^8–11^ Recent OCTA studies have reported a significant difference in the RNFL thickness and VD in the perimetrically intact regions of POAG eyes, when compared to the normal eyes.^10–12^

The purpose of this study is to evaluate the sectoral RNFL thickness and VD, using OCTA, in the eyes with unilateral PACG, with the VF defect, confined to the superior hemifield and compare it with the parameters in the opposite normal hemifield and corresponding normal hemifield in the fellow PAC eyes and normal control eyes.

## MATERIALS AND METHODS

This prospective, cross-sectional, observational study was conducted at our institute between July 2018 and October 2018. The study protocol was approved by the Institute Review Board of Anand Eye Institute, for research involving human subjects. Written informed consent was obtained from all the participants and the study was carried out in accordance with the tenets of the declaration of Helsinki.

All participants underwent a comprehensive ophthalmic examination, which included a detailed medical and ocular history, refraction, best-corrected visual acuity (BCVA) for distance and near, slit-lamp biomicroscopy, intraocular pressure (IOP) measurement with Goldmann applanation tonometry, gonioscopy with Sussman goniolens, dilated fundus evaluation, and SAP VF examination [Humphrey field analyzer (HFA) II, model 720i, Carl Zeiss Meditec, Dublin, CA], with the Swedish interactive threshold algorithm (SITA) standard 24-2 program. OCTA imaging of the optic disc and peripapillary region was performed with the RTVue-XR 100 Avanti OCT (version: A 2017,1,0, 151).

The study included 28 patients with unilateral PACG with the VF defect restricted to only superior hemisphere, 28 fellow eyes with PAC, and 30 control healthy eyes with the normal VFs on SAP. All PACG and PAC eyes had undergone laser peripheral iridotomy and were on anti-glaucoma medication (AGM) at the time of inclusion in the study. Pretreatment IOP, the IOP at which AGM was started, and the IOP at the time of obtaining OCTA were documented for all the eyes.

**Figs 1A to E:**
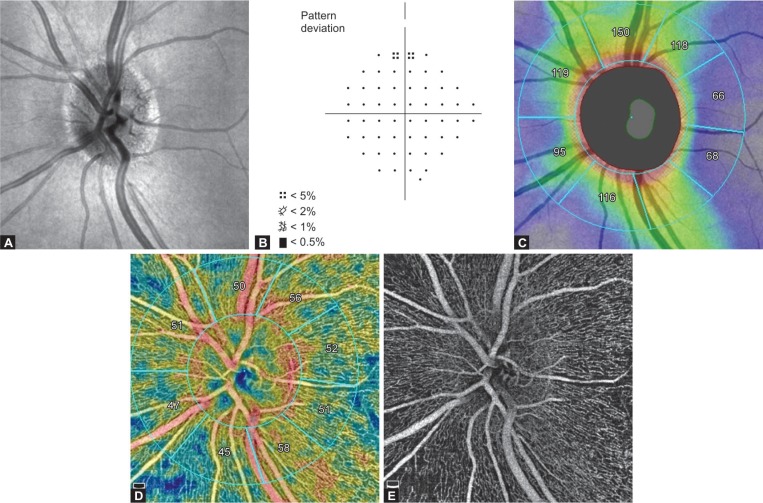
OCTA image of the optic disc in a normal eye; (A) Structural *en face* image of the healthy optic disc; (B) Humphrey VF analyzer 24-2 Swedish interactive threshold algorithm standard PDPP, shows intact superior and inferior hemifield; (C) Peripapillary RNFL measurement shows a normal RNFL thickness profile and the peripapillary region is divided into eight sectors based on the modified Garway-Heath of 2–4 mm grid. Inner ellipse indicates the optic disc boundary and the outer ellipse indicates the outer border of the peripapillary region; (D) OCTA scan of the peripapillary region showing VD of healthy eye. VD is measured in the sectors based on the modified Garway-Heath sector of 2–4 mm grid; (E) OCTA *en face* image shows dense radial peripapillary microvascular network around the optic disc

Normal subjects had no family history of glaucoma, IOP < 21 mm Hg, open angles on gonioscopy, normal anterior and posterior segments on the slit lamp examination, healthy optic discs (Fig. 1A), and normal VF on HVF 24-2, SITA standard. Normal VF indices were defined as the mean defect and corrected standard deviation within 95% confidence limits and a glaucoma hemifield test (GHT) result “within normal limits” and had no point worse than the 2% probability level in the pattern deviation probability plot (PDPP) (Fig. 1B).

Diagnosis of PAC disease was based on the definition proposed by the International Society for Geographical and Epidemiological Ophthalmology.^13^ PAC was defined as an eye in which there is an irido-trabecular contact for at least 270° on gonioscopy without indentation, using appropriate illumination, with raised IOP and/or peripheral anterior synechiae, normal optic disc and VFs. PACG was defined as PAC with evidence of glaucomatous optic disc changes (neuroretinal rim narrowing, rim notching, and RNFL defect) and corresponding VF changes.

PACG eyes had SAP VF defect restricted to the superior hemifield. VFs were considered reliable if the fixation losses were <20%, and the false-positive and false-negative response rates were <15%. The criteria for an abnormal VF included GHT results outside normal limits, pattern standard deviation (PSD) of probability <5% level, and the presence of a cluster of 3 or more contiguous, nonedge points in the typical glaucomatous locations that did not cross the horizontal meridian in the PDPP, with probability (*p*) < 5%, with at least one of them with a *p* value < 1%.^14^ The opposite normal hemifield had no point worse than 2% probability level in the PDPP.

Inclusion criteria for all the participants were age ≥40 years, distance BCVA of 20/30 or better, and refractive error within ±4 diopter (D) sphere and ±2 D cylinder. Exclusion criteria were a history of ocular trauma, intraocular surgery (other than cataract) presence of media opacities (that prevented good quality OCT scans), or any retinal or neurological disease.

### OCTA Data Acquisition Image Processing

OCTA imaging (AngioVue, AngioAnalytics v A 2017, 1, 0, 151) uses an 840-nm diode laser source, with an A-scan rate of 70 kHz per second. High-definition Angio disc has 400 B-scans, which are equally spaced on the *X*-axis and the *Y*-axis. Each B-scan has 400 A-scans, 640 pixels/A-scan, 400 A-scans/B-scan, and 400 B-scans/3D volume. OCTA uses the variation in OCT signal, caused by the moving particles, such as red blood cells, and compares the consecutive B-scans at the same location to detect blood flow, using motion contrast. Blood vessels are then delineated using the SSADA algorithm.^7^ VD is defined as the percentage of area occupied by the large vessels and microvasculature in a particular region and is calculated over the entire scan area, as well as in the defined sectors within the scan.

The Angio disc OCTA scan is performed using volumetric scans covering an area of 4.5 × 4.5 mm scan size. The software automatically fits an ellipse to the ONH margin. The peripapillary region is defined as a 1-mm wide elliptical annulus extending from the ONH boundary. This peripapillary region is divided into eight sectors [temporal upper (TU), supero-temporal (ST), superior nasal (SN), nasal upper (NU), nasal lower (NL), inferior nasal (IN), inferotemporal (IT), and temporal lower (TL)] based on the modified Garway-Heath sector of 2–4 mm grid (Fig. 1C). The software calculates peripapillary RNFL thickness and VD in each of these sectors and 2 hemispheres (superior and inferior). VD was calculated from the “RPC slab” which extends from the ILM to the posterior boundary of the RNFL (Fig. 1D).

For the ONH structural measurement, optic disc margin is automatically detected based on Bruch's membrane opening (BMO). Optic cup is defined by ILM segmentation line crossing below the BMO plane.

Image quality was assessed for all the OCTA scans (Fig. 1E). Poor quality scans (defined as those with a signal quality (SQ) score of <6), images with residual motion artifacts, and segmentation errors were excluded from the study.

### Statistical Analysis

Numeric data are presented as the mean and standard deviation. Independent 2-sample, Student *t* test was used for the group comparison of normally distributed variables; and Chi-square test was used to compare the categorical variables among the groups. Mean, hemisphere, and sector-wise OCTA-derived VD and RNFL thickness measurements were compared between the groups using ANOVA with the Bonferroni *post hoc* analysis. Statistical analysis was performed using the SPSS software (version 17, Inc., Chicago, IL, USA) and a *p* value < 0.05 was considered to be statistically significant.

## RESULTS

There was no significant difference among the age, gender, IOP at the time of scan, central corneal thickness, history of systemic hypertension, diabetes mellitus, and disc area between the PACG patients and normal controls (Table 1). As expected, the mean deviation and the pattern standard deviation were higher in PACG eyes when compared with the PAC and normal control eyes.

In the PACG eyes, there was a significant difference in the RNFL thickness (*p* < 0.0001) and RPC VD (*p* = 0.001) between the superior and inferior hemifield. In the PAC eyes, there was no significant difference in the RNFL thickness (*p* = 0.24) and RPC VD (*p* = 0.34) between the superior and the inferior hemifield. In normal eyes also, there was no significant difference in the RNFL thickness (*p* = 0.3) and RPC VD (*p* = 0.5) between the superior and the inferior hemifield.

**Table 1:** Baseline clinical characteristics of the study participants

*Variables*	*Primary angle-closure glaucoma eyes (n = 28)^A^*	*Fellow PAC eyes (n = 28)^B^*	*Normal controls (n = 30)^C^*	*p value (A vs B)*	*p value (A vs C)*	*p value (B vs C)*
Age (years)^€^	58.61 ± 11.48	58.61 ± 11.48	55.45 ± 13.78	–	0.5	0.5
Gender (Male : Female)^*^	12:16	12:16	14:16	–	0.18	0.18
Diabetes mellitus^*^	10	10	5	–	0.79	0.79
Systemic hypertension^*^	9	9	5	–	0.50	0.50
IOP in mm Hg (at the time of scan)^€^	14.55 ± 2.16	14.22 ± 2.13	14.19 ± 2.17	0.7	0.96	0.67
Mean deviation (decibel)^€^	−9.41 ± 4.71	−3.12 ± 1.22	−2.93 ± 1.85	<0.0001	<0.0001	0.88
Pattern standard deviation (decibel)^€^	8.19 ± 3.40	1.71 ± 0.68	1.81 ± 1.46	<0.0001	<0.0001	0.91
Central corneal thickness (microns)^€€^	512.5 ± 21.29	519.64 ± 23.63	519 ± 24.49	0.67	0.07	0.32
Disc area (mm^2^)^€^	1.81 ± 2.11	1.70 ± 2.1	1.88 ± 2.38	0.707	0.18	0.12

All values are represented as: mean ± standard deviation

* Chi-square test

€ Independent samples *t* test

IOP, Intraocular pressure

A: PACG eyes

B: Fellow PAC eye

C: Normal control

### PACG vs PAC Eyes

**Figs 2A to E:**
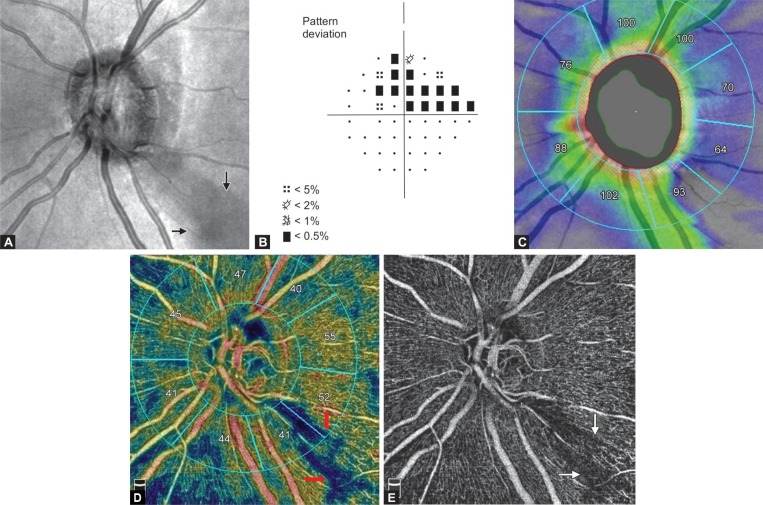
PACG eye with a superior hemifield defect; (A) OCTA structural *en face* image of the optic disc shows focal inferior temporal notch with corresponding RNFL defect at the peripapillary IT region (red arrows); (B) Humphrey VF analyzer 24-2 Swedish interactive threshold algorithm standard, PDPP shows a superior hemifield VF defect and normal inferior hemifield; (C) Peripapillary RNFL shows a reduced RNFL thickness at the IT and lower temporal sectors; (D) OCTA of PACG eye showing reduced VD in IT and lower temporal sectors; (E) OCTA *en face* image shows focal RPC dropout at the IT and lower temporal sectors

**Figs 3A to D:**
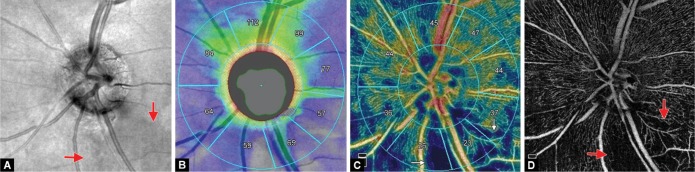
PACG eye with a superior hemifield defect; (A) OCTA structural *en face* image of optic disc shows focal inferior temporal notch with corresponding RNFL defect at the peripapillary IT region (red arrows); (B) Peripapillary RNFL shows reduced RNFL thickness in IT, lower temporal, and inferior nasal sectors; (C) OCTA of PACG eye showing reduced VD in IT, lower temporal, and inferior nasal sectors; (D) OCTA *en face* image shows wide RPC dropout at the IT, lower temporal, and inferior nasal sectors

In the PACG eyes with a inferior rim notch and corresponding RNFL defect (Figs 2A and 3A) and perimetrically affected superior regions (Fig. 2B), the RNFL thickness was reduced in IN, IT, and LT sectors (Table 2, Figs 2C and 3B) and there was a corresponding decrease in RPC VD (Table 2, Figs 2D and 3C) when compared with the PAC eyes. There was no difference in LN sector RNFL thickness (*p* = 0.2) and RPC VD (*p* = 0.13). Within the perimetrically intact regions of the PACG eyes, the mean RNFL thickness was significantly reduced in the SN and UN sectors (*p* = 0.02), but the VD did not show any significant difference, when compared with the fellow PAC eyes.

### PACG vs Normal Eyes

When compared to the normal eyes, RNFL thickness was reduced in the LN, IN, IT, and LT sectors of the perimetrically affected regions (Table 2) and there was a corresponding decrease in the RPC VD in the PACG eyes. The mean RNFL thickness was significantly reduced in the perimetrically normal SN and UN sectors (*p* < 0.0001) but the VD was significantly reduced only in the UN sector (*p* = 0.01) when compared with the normal eyes.

### PAC vs Normal Eyes

When comparing the peripapillary sectors of PAC and healthy eyes, the RNFL thickness was reduced in the UN (*p* = 0.02), LN (*p* = 0.01), IN (*p* = 0.02), and IT sectors (*p* = 0.03) and there was no significant difference in the VD in any of the sectors (Table 2).

In the normal eyes, the dense peripapillary microvascular network of RPC was observed around the ONH (Fig. 1E). In the PACG eyes with a hemifield defect, focal microvascular reduction or RPC dropout was seen at the corresponding sector of the RNFL defects (Figs 2E and 3D).

## DISCUSSION

In this study, OCTA was used for the quantitative evaluation of the structural OCT parameter (RNFL thickness) and peripapillary VD in the PACG eyes with VF defects, confined to the superior hemifield and compared it with the corresponding perimetrically normal sectors of the fellow PAC eye and healthy control eyes. As expected, the RNFL thickness and VD were reduced in the sectors corresponding to the hemifield defect in the PACG eyes, except in the LN sector. We also found that the RNFL thickness was decreased in the localized sectors of the perimetrically intact regions in the PACG and PAC eyes, when compared with the corresponding sectors in the normal eyes. On comparing PACG and PAC eyes, there was no significant difference in the VD in the perimetrically intact region. VD was reduced only in the UN sectors of PACG eyes when compared with the PAC eyes, in the perimetrically intact hemisphere.

**Table 2:** Hemifield and sector-wise analysis of peripapillary measurements of retinal nerve fiber layer thickness and vessel density as measured by optical coherence tomography angiography in primary angle-closure glaucoma eyes with superior hemifield defect, PAC fellow eyes and normal control eyes

	*PACG affecting superior hemifield (A)*	*PAC eyes (B)*	*Normal control (C)*	*Post hoc test, p value (A vs B)*	*Post hoc test, p value (A vs C)*	*Post hoc test, p value (B vs C)*
Signal quality	7.5 ± 1.32 (6.8–8.2)	7.67 ± 1.43 (6.75–8.58)	8.0 ± 1.25 (7.4–8.6)	0.742	0.27	0.497
RNFL thickness analysis (μm)						
Average	76.25 ± 9.99 (70.93–81.57)	95.33 ± 12.745 (87.24–103.43)	108.26 ± 12.476 (102.25–114.28)	<0.0001	<0.0001	0.005
Superior hemifield (180°)	87.88 ± 12.192 (81.38–94.37)	98.58 ± 13.548 (89.98–107.19)	110.37 ± 14.618 (103.32–117.41)	0.045	<0.0001	0.023
Inferior hemifield (180°)	63.88 ± 11.123 (57.95–69.80)	91.92 ± 13.681 (83.22–100.61)	105.95 ± 11.336 (100.48–111.41)	<0.0001	<0.0001	0.003
Sector wise RNFL thickness analysis (μm)						
Superior nasal	106.25 ± 23.254 (93.86–118.64)	127.50 ± 24.843 (111.72–143.28)	137.79 ± 22.087 (127.14–148.44)	0.021	<0.0001	0.235
Upper nasal	79.75 ± 13.409 (72.60–86.90)	95.92 ± 17.962 (84.50–107.33)	111.58 ± 18.680 (102.58–120.58)	0.016	<0.0001	0.016
Lower nasal	64.63 ± 14.099 (57.11–72.14)	72.17 ± 12.467 (64.25–80.09)	88.00 ± 17.591 (79.52–96.48)	0.203	<0.0001	0.007
Inferior nasal	72.44 ± 21.584 (60.94–83.94)	114.58 ± 24.243 (99.18–129.99)	134.84 ± 24.377 (123.09–146.59)	<0.0001	<0.0001	0.024
Inferior temporal	61.81 ± 18.276 (52.07–71.55)	116.08 ± 21.420 (102.47–129.69)	131.68 ± 17.77 (123.12–140.25)	<0.0001	<0.0001	0.03
Lower temporal	54.50 ± 12.946 (47.60–61.40)	66.00 ± 10.946 (59.05–72.95)	69.79 ± 12.259 (63.88–75.70)	0.017	0.001	0.404
Upper temporal	65.31 ± 13.119 (58.32–72.30)	72.50 ± 19.515 (60.10–84.90)	72.42 ± 13.031 (66.14–78.70)	0.215	0.168	0.989
Superior temporal	105.81 ± 17.065 (96.72–114.91)	112.50 ± 30.138 (93.35–131.65)	120.21 ± 27.381 (107.01–133.41)	0.49	0.099	0.41
OCTA RPC vessel density (%)						
Superior hemifield (180°)	48.19 ± 5.776 (45.11–51.27)	51.58 ± 4.252 (48.88–54.29)	52.26 ± 3.016 (50.81–53.72)	0.051	0.01	0.679
Inferior hemifield (180°)	39.00 ± 7.780 (34.85–43.15)	49.42 ± 6.417 (45.34–53.49)	51.68 ± 2.868 (50.30–53.07)	<0.0001	<0.0001	0.299
Sector wise OCTA RPC vessel density (%)						
Superior nasal	46.63 ± 8.156 (42.28–50.97)	49.67 ± 5.598 (46.11–53.22)	49.53 ± 5.243 (47.00–52.05)	0.224	0.193	0.953
Upper nasal	44.69 ± 6.364 (41.30–48.08)	48.67 ± 4.774 (45.63–51.70)	49.58 ± 4.799 (47.27–51.89)	0.059	0.01	0.648
Lower nasal	41.69 ± 6.730 (38.10–45.27)	45.17 ± 6.043 (41.33–49.01)	47.95 ± 5.169 (45.46–50.44)	0.133	0.003	0.212
Inferior nasal	35.19 ± 13.014 (28.25–42.12)	47.25 ± 7.557 (42.45–52.05)	50.26 ± 5.075 (47.82–52.71)	0.001	<0.0001	0.373
Inferior temporal	32.06 ± 9.630 (26.93–37.19)	56.75 ± 10.584 (50.03–63.47)	57.37 ± 4.349 (55.27–59.46)	<0.0001	<0.0001	0.839
Lower temporal	45.50 ± 7.840 (41.32–49.68)	50.33 ± 6.228 (46.38–54.29)	52.05 ± 2.915 (50.65–53.46)	0.036	0.002	0.429
Upper temporal	51.00 ± 7.439 (47.04–54.96)	55.25 ± 5.941 (51.48–59.02)	54.84 ± 3.132 (53.33–56.35)	0.054	0.05	0.845
Superior temporal	51.94 ± 7.289 (48.05–55.82)	55.17 ± 9.094 (49.39–60.94)	56.11 ± 3.999 (54.18–58.03)	0.216	0.075	0.707

All values represent mean ± standard deviation with 95% confidence intervals (lower bound and upper bound) for mean in parenthesis

OCTA, optical coherence tomography Angiography

PACG, primary angle closure glaucoma

RNFL, retinal nerve fiber layer

RPC, radial peripapillary capillary

A: PACG eyes

B: PAC eyes

C: Normal control

With optical microangiography, Chen et al.^9^ showed reduced peripapillary microcirculation in the normal hemisphere of 21 POAG eyes with hemifield defects. They found no significant difference in the RNFL thickness between the perimetrically intact regions of POAG and 20 healthy controls and concluded that the vascular changes precede structural and functional changes in glaucoma. This is contradictory to the other reports;^10–12^ however, in their study, normal subjects did not undergo HFA and the sectoral peripapillary VD was not analyzed.

The OCTA study by Yarmohammadi et al.^10^ found a reduced peripapillary RNFL thickness and VD in the perimetrically intact hemifield in 58 POAG eyes when compared with the 28 healthy eyes and concluded that the structural and vascular changes precede functional damage in glaucoma. In their study also, sectoral peripapillary VD was not measured.

Using OCTA (optic disc scan protocol: 3 × 3 mm), Akagi et al.^11^ reported that the peripapillary RNFL (defined as a 500-μm elliptical annulus around the disc) thickness is significantly thinner in the perimetrically intact region and found no difference in the peripapillary VD between the perimetrically intact regions of POAG and healthy eyes. They suggested that the RNFL thinning precedes vascular changes and functional loss. These findings are similar to what was observed in our study on the PACG eyes, despite certain differences, i.e., the older version of the AngioVue was used in their study, the peripapillary region was divided into six sectors based on Garway-Heath map, and the VD was measured from ILM extending up to 100 μm below the ILM.

Pradhan et al.^12^ studied sectoral VD and RNFL thickness in 31 POAG eyes with the VF defect restricted to one hemifield (24 eyes with superior hemifield defect and 7 had inferior hemifield defect) and compared it with the 41 age-matched control eyes using OCTA imaging (AngioVue, v2015.100.0.33, scan protocol 4.5 × 4.5 mm) for VD measurement. In the POAG eyes, the VD was reduced in the TU, SN, NU, and IT sectors and the RNFL was decreased in the ST, SN, NU, and IT sectors, despite being perimetrically normal. They concluded that the peripapillary vascular changes precede functional decline. Compared to our study, an older version of the AngioVue OCTA was used in their study and the peripapillary region was defined as a 0.75-mm annulus around the ONH, and the RNFL thickness was measured by RTVue-XR SD-OCT along a circle diameter of 3.45 mm, centered on the optic disc. In our study, the peripapillary region was defined as a 1-mm wide elliptical annulus around the optic disc and the RNFL thickness and VD were measured in the same corresponding 8 peripapillary sectors, all based on the Garway-Heath grid areas. All these OCTA differences in the parameters measured may have caused the difference in the study outcome.

In our study, sector analysis showed a reduced RNFL thickness in the SN and UN sectors of the perimetrically intact PACG eyes when compared to PAC and normal eyes and reduced RPC VD in only the UN sector of PACG eyes, when compared to the normal. On comparing PAC eyes and normal controls, RNFLT was reduced in the UN, LN, IN, and IT sectors, while the RPC VD did not show a significant difference in any of the sectors. This interesting observation shows that the reduction in peripapillary RNFL thickness precedes VD reduction and VF loss on SAP in PACG eyes with hemifield VF defect. Also, in the PAC eyes, there was a significant reduction in the RNFL thickness in selective sectors which could be due to an increase in IOP, which has preceded the functional loss as seen in the perimetrically normal hemifield. Probably, if these PAC eyes are not treated, they may be at a risk of progressive RNFL loss resulting in the perimetrically apparent functional visual loss and it also implies that the decrease in the VD seems to be a consequence of RNFL thinning.

In the perimetrically affected superior hemisphere of PACG eyes, LN sector did not show a decrease in RNFL thickness and VD. The reason for this could be that there is a wide variation in RNFL defect and VF loss among PACG eyes. Some eyes had a focal notch, localized, narrow RNFL defect with early VF loss, and a lesser degree of RNFL reduction (Fig. 2), while some had wider notching with a wide wedge-shaped RNFL defect with more VF damage and a greater degree of RNFL thinning and VD reduction (Fig. 3).

The strength of our study is that we have used a strict criterion to define normal VF in the PAC, healthy eyes and perimetrically normal hemifield of PACG eyes, which ensured that the normal regions without functional deficit were evaluated. SQ is known to have a significant positive association with peripapillary VD.^15^ Some of the previous OCTA studies^9,11^ have not performed VFs in the healthy controls and have not compared SQ between the glaucomatous and healthy eyes. In our study, only high-quality OCTA images (SQ > 6) were included and there was no significant difference between SQ of the OCTA images between the groups. Also, while comparing RNFL thickness and VD, the SQ was the same for both the parameters and the sector measurement was made in the corresponding area as opposed to the previous studies, where the VD was compared to the RNFL thickness, measured in 45° equally divided sectors.^10–12^

The present study has a few limitations. First, as we observed that the number of PACG eyes (*n* = 3) with VF defects limited to the inferior hemifield was small, we restricted the study to only the eyes with superior VF defects. Second, the effect of systemic antihypertensive and AGM on the VD parameters was not assessed, and we did not exclude patients with other vascular diseases like diabetes and hypertension. However, these parameters were well matched between the two groups (Table 1) and, therefore, any difference in VD is likely to be due to the disease itself. Third, being a cross-sectional study, we cannot comment on the cause–effect relationship between the VD and RNFL thickness.

In conclusion, peripapillary structural changes precede vascular loss and functional deficit in PACG, as the sectors that have no VF defect in PACG eyes already demonstrate RNFL thinning on OCT. In PAC eyes, RNFL thinning and VD loss precedes functional deficit. The extent of VD reduction and RNFL thinning varies in different peripapillary sectors. Longitudinal studies with a larger sample size may help to better understand the temporal relationship of RNFL thickness and VD loss in the PACG and PAC eyes.

## CONCLUSION

We studied peripapillary RNFL thickness and RPC VD in the eyes with unilateral PACG with the VF defect confined to the superior hemifield and found that the RNFL thinning precedes vascular changes and functional loss in the PACG eyes.

## References

[1] Quigley HA,, Addicks EM, (1982;). Optic nerve damage in human glaucoma. III. Quantitative correlation of nerve fiber loss and visual field defect in glaucoma, ischemic neuropathy, papilledema, and toxic neuropathy.. Arch Ophthalmol.

[2] Rao HL,, Kadambi SV, (2017;). Diagnostic ability of peripapillary vessel density measurements of optical coherence tomography angiography in primary open-angle and angle-closure glaucoma.. Br J Ophthalmol.

[3] Mansoori T,, Sivaswamy J, (2017;). Radial Peripapillary Capillary Density Measurement Using Optical Coherence Tomography Angiography in Early Glaucoma.. J Glaucoma.

[4] Liu L,, Jia Y, (2015;). Optical Coherence Tomography Angiography of the Peripapillary Retina in Glaucoma.. JAMA Ophthalmol.

[5] Jia Y,, Morrison JC, (2012;). Quantitative OCT angiography of optic nerve head blood flow.. Biomed Opt Express.

[6] Mansoori T,, Sivaswamy J, (2017;). Measurement of Radial Peripapillary Capillary Density in the Normal Human Retina Using Optical Coherence Tomography Angiography.. J Glaucoma.

[7] Jia Y,, Tan O, (2012;). Split-spectrum amplitude decorrelation angiography with optical coherence tomography.. Opt Express.

[8] Rao HL,, Pradhan ZS, (2016;). Regional Comparisons of Optical Coherence Tomography Angiography Vessel Density in Primary Open-Angle Glaucoma.. Am J Ophthalmol.

[9] Chen C,, Bojikian KD, (2017;). Peripapillary Retinal Nerve Fiber Layer Vascular Microcirculation in Eye with Glaucoma and Single-Hemifield Visual Field Loss.. JAMA Ophthalmol.

[10] Yarmohammadi A,, Zangwill LM, (2017;). Peripapillary and Macular Vessel Density in Patients with Glaucoma and Single-Hemifield Visual Field Defect.. Ophthalmology.

[11] Akagi T,, Iida Y, (2016;). Microvascular Density in Glaucomatous Eyes with Hemifield Visual Field Defects: An Optical Coherence Tomography Angiography Study.. Am J Ophthalmol.

[12] Pradhan ZS,, Dixit S, (2018;). A Sectoral Analysis of Vessel Density Measurements in Perimetrically Intact Regions of Glaucomatous Eyes: An Optical Coherence Tomography Angiography Study.. J Glaucoma.

[13] Foster PJ,, Buhrmann R, (2002;). The definition and classification of glaucoma in prevalence surveys.. Br J Ophthalmol.

[14] Anderson DR,, Patella VM. (1999.). Automated static perimetry,.

[15] Rao HL,, Pradhan ZS, (2017;). Determinants of Peripapillary and Macular Vessel Densities Measured by Optical Coherence Tomography Angiography in Normal Eyes.. J Glaucoma.

